# Are women at a higher risk of dementia? A National Study of Underlying Background Medical Conditions

**DOI:** 10.1002/alz.088013

**Published:** 2025-01-09

**Authors:** Anat Rotstein, Arad Kodesh, Stephen Z Levine

**Affiliations:** ^1^ University of Haifa, Haifa Israel; ^2^ Meuhedet Health Maintenance Organization, Tel Aviv Israel

## Abstract

**Background:**

Studies of the association between sex and the risk of incident dementia are inconsistent, in part conceivably due to sex‐specific background medical conditions.

**Method:**

A prospective national cohort study which consisted of 112,204 members of a nonprofit Israeli health maintenance organization born between 1902 and 1952 who entered the cohort on January 1, 2002, without a dementia diagnosis and were followed up to January 1, 2020. Participants were aged 50 to 100 years. Incident dementia was ascertained based on medical diagnoses with International Classification of Diseases codes from the Ninth (331.0‐331.9) and Tenth (F00–F03) revisions. Cox regression models were fitted to quantify the association between sex and the risk of incident dementia with Hazard Ratios (HR) and their 95% Confidence Intervals (CI) unadjusted and adjusted for 34 sources of potential confounding.

**Results:**

Women had a higher unadjusted risk for dementia. After adjustment for two demographic factors (birth year and socioeconomic status) and 32 medical conditions (psychiatric disorders, musculoskeletal system, and connective tissue disorders, metabolic syndromes, cardiovascular diseases, respiratory diseases, neurological diseases, genitourinary, digestive, malignant diseases, and injuries) the association between sex and dementia was no longer significant.

**Conclusion:**

The commonly reported increased dementia risk for women does not extend once a history of selected medical conditions is considered, suggesting that a more focused outlook on the association between sex and dementia may be a step forward in the identification of at‐risk groups and dementia prevention.

